# Advances in Hybrid Brain-Computer Interfaces: Principles, Design, and Applications

**DOI:** 10.1155/2019/3807670

**Published:** 2019-10-08

**Authors:** Zina Li, Shuqing Zhang, Jiahui Pan

**Affiliations:** South China Normal University, Guangzhou 510631, China

## Abstract

Conventional brain-computer interface (BCI) systems have been facing two fundamental challenges: the lack of high detection performance and the control command problem. To this end, the researchers have proposed a hybrid brain-computer interface (hBCI) to address these challenges. This paper mainly discusses the research progress of hBCI and reviews three types of hBCI, namely, hBCI based on multiple brain models, multisensory hBCI, and hBCI based on multimodal signals. By analyzing the general principles, paradigm designs, experimental results, advantages, and applications of the latest hBCI system, we found that using hBCI technology can improve the detection performance of BCI and achieve multidegree/multifunctional control, which is significantly superior to single-mode BCIs.

## 1. Introduction

Brain-computer interface (BCI) is a technology that translates signals generated by brain activity into control signals without the involvement of peripheral nerves and muscles and uses these signals to control external devices [1]. In recent years, BCI has attracted increasing attention from academia and the public due to its potential clinical application. For example, BCI can provide augmented or repaired motor function, which can be of great help to patients with severe motor impairment. The most commonly used methods of extracting brain signals are nonimplanting, including functional magnetic resonance imaging (fMRI), magnetoencephalography (MEG), electroencephalography (EEG), and functional near-infrared spectroscopy (fNIRS) [2]. Although EEG has low signal-to-noise ratio and spatial resolution, it has been widely used in BCI because of its noninvasiveness, portability, low cost, good performance, real-time response, and technical requirements lower than other brain signals. This paper mainly describes the BCI based on EEG. Brain models used in EEG-based hybrid BCIs typically include the P300 visual-evoked potential proposed by Farwell and Donchin in 1988 [3], the steady-state-evoked potential (such as the steady-state visual-evoked potential (SSVEP)) [4] and event-related desynchronization/synchronization (ERD/ERS) generated by motor imagination (MI) [5].

Conventional EEG-based BCI generally relies solely on a single-signal input (such as EEG, electromyography (EMG), and electro-oculogram (EOG)), single sensory stimulus (such as visual only, auditory only, and tactile only), or single brain pattern (such as the above P300 potential and SSVEP). The single-mode BCI system has achieved great progress in paradigm design, brain signal processing algorithms, and applications. However, these BCI systems have been facing multiple challenges, including low information transfer rates (ITRs), low man-machine adaptability, and high dynamics/nonstationarity of brain signals [6, 7]. Here, we mainly consider two fundamental challenges and introduce a hybrid BCI technique intended to address these challenges:

1. Multidegree/multifunction control: multidegree/multifunctional control is necessary for many devices, such as wheelchair, robots, or artificial limbs. For instance, the wheelchair control includes speed, direction, and start/stop functions. However, it is difficult for a conventional simple BCI to generate effective multiple control signals [8].
2. Improvement of detection performance: over the years, although many efforts have been made to improve the detection performance of BCI, the detection performance in terms of classification accuracy, information transfer rate (ITR), and false-positive rate (FPR) is still far from practice in many applications, especially for patients. Approximately 13% of healthy users suffer from BCI illiteracy and do not reach the criterion for controlling a BCI application [9]. Moreover, user acceptability and complexity of the BCI systems should be reported as important performance criteria.

To conquer the above two fundamental challenges, some researchers have proposed a hybrid BCI (hBCI). As described by Allison [8], an hBCI system consists of a BCI system and an add-on system, which can be a second BCI system, but designed to perform specific goals better than a conventional BCI. The main goal of hBCI is to overcome the existing limitations and disadvantages of the conventional BCI systems. In this paper, the recent progress in hBCIs was reviewed to illustrate how hBCI techniques could be implemented to address these challenges. The definition of hybrid BCIs was updated and extended, and three main types of hBCIs have been devised. For each type of hybrid BCIs, the principle was summarized and several representative hybrid BCI systems were highlighted by analyzing their paradigm designs, control methods, and experimental results. Finally, the future prospect and research direction of hBCI were discussed.

## 2. Hybrid BCI Overview

Although the concept of hBCI emerged before 2010, its development has become more and more rapid in recent years. Based on the search engine “Web of Science,” and title-abstract-keyword ((“brain-computer interface” or “BCI”) and (“hybrid” or “multimodal”), the number of journal papers found before 2010 was only three. However, this number rose to 148 and 293 in the two periods of 2010–2014 and 2015–2019, respectively. It is evident that the number of publications on hBCI has grown rapidly in recent years. Note that those studies of single BCI combining only features and algorithms also can improve performance are excluded. In fact, “Hybrid BCI” and “multimodal BCI” are two highly related concepts. Li et al. [9] even considered that “hybrid BCI” and “multimodal BCI” to be interchangeable terms with the same BCI definition.

Pfurtscheller et al. [10] believed that in addition to the simple combination of different BCIs, the type of hBCI should meet the following four criteria: (1) the activity comes directly from the brain; (2) at least one brain signal acquisition method should be used to capture this activity, and the brain signal acquisition method can be in the form of electrical, magnetic, or hemodynamic changes; (3) the signal must be processed in real time/online to establish communication between the brain and the computer to generate control commands; (4) feedback must be provided according to the results of brain activity for communication and control.

**Figure 1:**
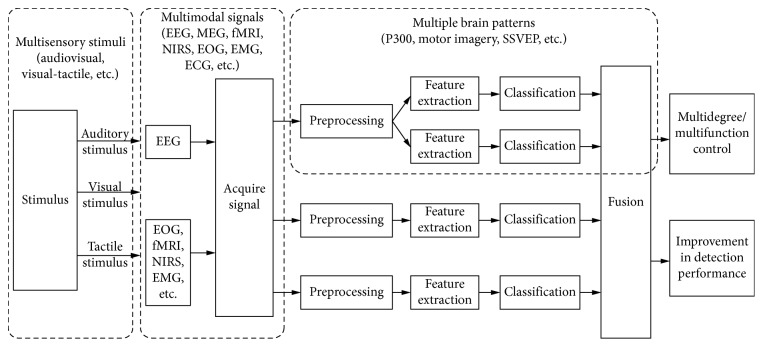
The signal flow of hybrid brain-computer interface discussed in this paper.

The signal flow of an hBCI system is as described in Figure 1, which includes two stages of brain signal processing. (1) In the signal acquisition, the signal input can be from multiple signals (e.g., EEG and NIRS) or multiple brain patterns (e.g., P300 and SSVEP), which are evoked by multisensory stimuli (e.g., audiovisual stimuli). (2) In the signal processing, an hBCI system can provide only a single-output/control signal or multiple-output/control signals. In the former case, when multiple brain patterns or multiple signals are involved, data fusion is generally required at the feature or decision level. In the latter case, multiple control signals may be separately manipulated by different brain patterns detected by the system, and the fusion of these brain patterns is generally not necessary. As shown in Figure 1, the hBCI can be divided into three main categories:

1. hBCI based on multiple brain patterns: it uses at least two brain modes (e.g., P300 and SSVEP or MI and P300). In this type of hBCI, multiple brain patterns are induced by a single sensory stimulus. Several studies have indicated that hybrid integration associated with multimodal stimuli has the potential to enhance brain patterns, which may be beneficial for BCI performance [11].
2. hBCI with multisensory stimuli: its brain pattern is simultaneously induced by multiple sensory stimuli, such as audiovisual stimuli. In this hBCI, one or more brain patterns are induced by multisensory stimuli. Some researchers believed multisensory BCIs may offer more versatile and user-friendly paradigms for control and feedback [12].
3. hBCI based on multiple signals: in this hBCI, two or more input signals are typically combined with a hybrid BCI system, such as EEG, MEG, fMRI, fNIRS, EOG, or EMG. Different brain signals have different signal characteristics and can be used for different functions.

The state-of-the-art of the above three types of hBCI is introduced in the following sections, including their general principles, stimuli paradigm, control methods, corresponding experimental results, and advantages.

## 3. hBCI Based on Multiple Brain Patterns

The first class of hBCIs combines multiple brain patterns, such as P300, SSVEP, and MI. It has been designed for a variety of applications, such as speller [13], idle state detection [14], orthotics [15], the wheelchair navigation, and control of computer components, which include two-dimensional (2D) cursor [16], mouse [17], or mail client [18].  Table 1 lists the representative hBCI applications of multiple brain patterns in recent years. In this section, we mainly describe hBCI based on P300 and SSVEP, hBCI based on MI and SSVEP, and hBCI based on MI and P300.

### 3.1. P300- and SSVEP-Based hBCIs

Both P300 potential and SSVEP can be elicited by visual stimuli, allowing subjects to evoke both brain patterns by performing a visual attention task without extra mental load. The P300 and SSVEP features are located in different domains (time domain versus frequency domain), and both brain patterns have significant independence. The improvement in performance may result from the utilization of both P300 and SSVEP features. The addition of the EEG feature may provide additional information that facilitates the classification of a target versus a nontarget.

Bi et al. [22] proposed a hybrid paradigm based on SSVEP and P300 for developing speed-direction-based cursor control. In this study, the stimulation of the P300 was distributed on the upper and lower sides of the screen, and the stimulus for detecting SSVEP (which can rotate the control device clockwise or counterclockwise) was displayed on the left and right sides of the screen. The results using the method based on the support vector machine classification showed that the accuracy of the hBCI was higher than 90%.

Pan et al. [29] detected consciousness in eight patients with disorders of consciousness (DOC) by using a hybrid paradigm of SSVEP and P300. Following the instructions, the left- and right-hand photos flickered on a black background with fixed frequencies of 6.0 and 7.5 Hz, respectively, to evoke the patient's SSVEP. Meanwhile, each of the two photo frames was randomly presented five times to evoke P300, with each appearance lasting 200 ms and the interval between two consecutive appearances being 800 ms. The BCI system used the characteristics of P300 and SSVEP to detect which photo the patient had noticed. Eight patients (four in the vegetative state (VS), three in the minimally conscious state (MCS), and one in the locked-in syndrome (LIS)) participated in the experiment. Using the SVM-based classifier, one VS patient, one MCS patient, and one LIS patient were able to select photos of themselves or others (classification accuracy, 66%–100%), which indicates that the patient command can be followed using an hybrid BCI and further proves that they have certain cognitive abilities and awareness.

### 3.2. MI- and SSVEP-Based hBCIs

There are four reasons to combine SSVEP and MI: (1) SSVEP- and MI-related brain patterns were produced simultaneously; (2) SSVEP is an evoked potential that can be stably detected in unfamiliar subjects with little training, but for most new users, it is difficult to adapt to the process of completing MI task; (3) SSVEP can detect by a single trial based on EEG data, and the detection does not require an averaging process; (4) nonvisual training will frustrate subjects, while SSVEP provides a possible solution to attract subjects to participate in MI task.

Based on the above principles, Yu et al. [26] combined SSVEP and MI to provide effective continuous feedback for MI training in 24 subjects. Initially, the classifier assigns a greater weight to the SSVEP in order to get the correct feedback at the beginning of the training. As the training goes on, participants reduced their visual attention to SSVEP stimuli but maintained sustained attention to MI mental tasks. When subjects adapt to rhythmic activities, the classifier shifts the weight to MI. The result showed that an hBCI can be used to improve MI training and produce distinguishable brain patterns after only five sessions (about 1.5 hours).

### 3.3. MI- and P300-Based hBCIs

An important aspect of the EEG-based BCI system is multidimensional control, which involves multiple independent control signals. These control signals can be obtained from multiple brain patterns, such as MI and P300. P300 represents the reliable type of brain pattern used to generate discrete control output commands, and MI is more effective against generating sequential control commands.

**Figure 2:**
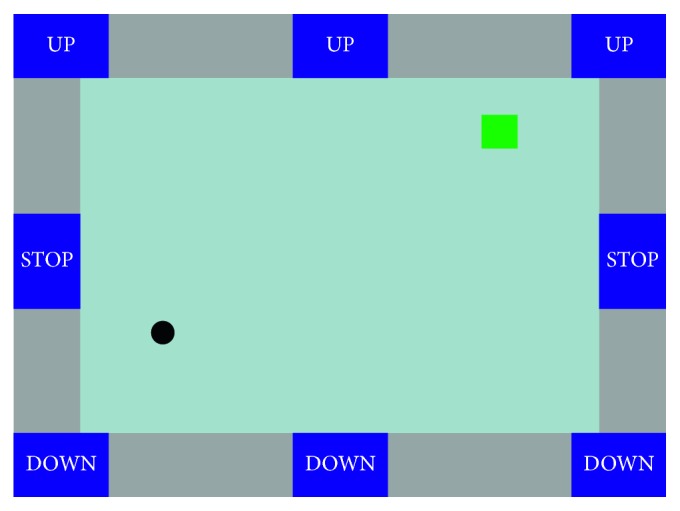
GUI of 2D cursor control and target selection of a hBCI system [16], which combines MI and P300 potential, including one cursor (black circle), one object (green square), and eight flashing buttons (three “UP,” three “DOWN,” and two “STOP” buttons).

Li and colleagues [16] proposed hBCI combining MI brain patterns and P300 potentials for 2D cursor control and target selection. The GUI is shown in Figure 2, in which the circle and square represent the cursor and target, respectively, with the initial position of the cursor and the initial position and color (green or blue) of the target are randomly provided. The three “UP” buttons, three “DOWN” buttons, and two “STOP” buttons flash in a random order to evoke P300 potentials. The task of the user is to move the cursor to the target and then to select or reject the green/blue target. The control strategy of the user is described below. The user can move the cursor to the left or right by imagining his or her own left- or right-hand movement, respectively, and the user can move the cursor up or down by focusing on one of the three flashing “UP” or “DOWN” buttons to evoke P300 potentials. If the user does not intend to move the cursor in the vertical direction, then the user can focus on one of the two “STOP” buttons.

To further implement a BCI mouse, target selection and rejection functions are required. Specifically, once the cursor hits the target of interest (green square), the user can select the target by focusing the attention on a flashing “STOP” button and simultaneously maintaining an idle state of motor imagery. If the target is not of interest (blue square), the user can reject it by continuing to imagine left- or right-hand movement without focusing on any flashing buttons.

The algorithm for the 2D cursor control includes two parts: P300 detection for vertical movement control and motor-imagery detection for horizontal movement control, with the details presented in [19]. The signal processing procedure for P300 detection consists of three stages: low-pass filtering, P300 feature extraction, and SVM classification. For motor-imagery detection, the signal processing stages include common average reference (CAR) spatial filtering, band-pass filtering of the specific mu rhythm band (8–13 Hz), feature extraction based on a CSP algorithm, and SVM classification. The algorithm for target selection or rejection was based on the hybrid features of P300 potentials and MI. After extracting the features of the P300 potentials and MI using the same algorithms described above, a hybrid feature vector for each trial is constructed by concatenating the feature vector of the MI with the feature vector of the P300 potentials, which is then fed into the SVM for classification.

Eleven healthy subjects attended the online experiment, which included one session of 80 trials for each subject. Each trial included two sequential tasks. During the first task, subjects were instructed to move the cursor to a target that was presented at a randomized position on the screen. After the cursor hit the target, the subject was instructed to perform the second task of selecting or rejecting the target according to the color of the target (green for selection and blue for rejection). The time interval for the second task was set to 2 s. Among all subjects, the average time for one trial was 18.96 s, the average accuracy for successful trials was 92.84%, and the average for target selection accuracy given that the cursor was successfully moved to the target was 93.99%. Additionally, several datasets were also collected for offline analysis to demonstrate the advantage of P300 potential and MI hybrid features for target selection/rejection compared with the use of P300 potential or MI features alone. The experimental results showed that the accuracy for use of the hybrid features was significantly higher than for use of only the MI or P300 potential features (hybrid features: 83.10 ± 2.12%; MI features: 71.68 ± 2.41%; P300 features: 80.44 ± 1.82%). Based on the BCI cursor, Long et al. [28] proposed a hybrid BCI paradigm based on MI and P300 potential to operate actual wheelchairs by providing direction (left or right) and speed control (acceleration and deceleration) commands with 5 subjects.

All of these hybrid systems have three advantages. First, two independent control signals are generated based on MI and P300 potential. Second, the user can move the cursor from any position to a randomly located target. Third, the hybrid control strategy using MI and P300 potential provides better identification performance than the control strategy using MI-only or P300-only.

## 4. Multisensory hBCIs

Humans have multiple senses that provide pathways for processing information on the reality. The integration of multiple sensory stimuli enhances top-down attention, and these enhanced effects may be conducive to improve the performance of BCI systems. Taken into this consideration, hBCI based on audiovisual and visual-tactile was proposed, in which bimodal stimulation was used to improve system performance.  Table 2 lists the representative applications of multisensory hBCIs in recent years.

### 4.1. Audiovisual hBCIs

Belitski et al. [30] proposed an offline audiovisual-based P300 speller and corresponding data analysis results. Their study of 7 healthy subjects showed that the intensity of P300 reaction was higher in audiovisual conditions than in visual or auditory conditions alone. Similarly, An et al. [32] explored parallel spellers for BCI unrelated to gaze for healthy subjects, where the auditory and visual domains are independent of each other. Their results showed that 15 users can spell online, with an average accuracy rate of 87.7%. These existing results suggest that audiovisual integration may be a potential way to enhance brain patterns and further improve BCI performance. Wang et al. [33] proposed a novel audiovisual BCI system, which is based on time-synchronous visual and auditory stimuli. In the GUI of this audiovisual BCI, there are two number buttons (two numbers randomly drawn from 0 to 9) located on the left and right sides, and two speakers are placed laterally to the monitor. The two buttons flash in an alternative manner. When a number button is visually intensified, the corresponding spoken number is presented from the ipsilateral speaker. In this way, the user is presented with a temporally, spatially, and semantically congruent audiovisual stimulus that lasts for 300 ms, where the interstimulus interval is randomized from 700 to 1500 ms. Ten healthy subjects participated in the experiment. The experiment consisted of three sessions administered in a random order, corresponding to the visual-only, auditory-only, and audiovisual conditions. In each session, the subject first performed a training run of 10 trials and then a test run of 30 trials. The online average accuracy of audiovisual, visual-only, and auditory-only sessions for all healthy subjects was 95.67%, 86.33%, and 62.33%, respectively. The audiovisual BCI significantly outperformed the visual-only and auditory-only BCIs. This audiovisual hBCI system was then applied to the consciousness detection of 7 patients with DOC. The experimental results indicated that the audiovisual BCI can provide more sensitive results than the behavioral observation scale.

### 4.2. Audio-Tactile hBCIs

The above bimodal BCI requires visual interaction to focus on stimuli and feedback, which limits their applicability to users with good vision and complete gaze control. Since the user does not require visual interaction when operating auditory or tactile BCI, a bimodal auditory/tactile-based manner may allow visual scanning of unrelated BCI. Yin et al. [34] proposed a dual-mode P300 BCI with the same direction, which was presented simultaneously with auditory and tactile stimuli from the same spatial direction. Rutkowski and Mori [35] studied the tactile and auditory BCI of 11 users with vision and hearing impairment.

These existing results reveal the several advantages of BCI auditory-tactile. First, the auditory-tactile dual-mode BCI has better overall system performance than the auditory or tactile single-mode P300 BCI. Second, in visual computer applications, auditory-tactile hBCI offers an attractive possibility of target sensory fields that can induce potential without relying on visual stimuli, although the performance achieved by using this system is lower than that of BCI dependent on gaze transfer. Third, visual-tactile hBCI is an alternative for users with impaired vision.

## 5. hBCI Based on Multimodal Signals

hBCI systems can be constructed using multimodal signals, including EEG, MEG, fMRI, EOG, fNIRS, and EMG. Different brain signals have different signal characteristics and can be used for different functions. Recently, several hybrid BCIs based on multiple signals have been reported in the following.  Table 3 lists the representative hBCI applications based on multimodal signals in recent years.

### 5.1. EEG- and EMG-Based hBCIs

Leeb et al. [50] proposed an hBCI combining EEG and EMG. In each trial, 12 healthy subjects were instructed to repeat the exercise for five seconds with their left or right hand (holding the hand with the fist) based on visual cues (arrows to the left or right). The researchers processed and classified EEG and EMG signals separately and then fused them. Canonical variable analysis was used to select subject-specific features that maximized separability between different tasks, and stable features were determined by cross validation of a Gaussian classifier based on training data. The resulting features were given threshold, normalized, and classified based on maximum distance in a subject-specific manner. Finally, the Bayesian method was used to fuse the probabilities of two classifiers to generate a control signal. The accuracy of a single EEG activity was 73% and single EMG activity was 87%. However, the accuracy of the hBCI was improved to 91%. In addition, to simulate tired muscles, the amplitude of the EMG channel decreased during operation (from 10% to 100%), and EEG activity is increasingly important in fused data as EMG muscles become more tired. The results showed a significant advantage for EEG- and EMG-based BCI systems.

### 5.2. EEG- and EOG-Based hBCIs

Recently, some studies have combined EEG and EOG to construct an hBCI. Since many people with disabilities are able to control their eye movements, EOG signals are an appropriate choice for many users of the BCI system. Lee et al. [41] employed hBCIs based on EEG-EOG to a speller system with fast typing speed. The hBCI system comprised a conventional ERP-based speller, an EOG-based command detector, and a visual feedback module. The online ERP speller was used to compute the classification probabilities for all candidate characters from EEG epoch. The character of highest probability was selected as visual feedback based on the probabilities sorting. The accuracy of the novel speller system was 97.6%, and its ITR is 39.6 ± 13.2 bits/min across 20 participants. The result showed that this EEG- and EOG-based speller has better performance than the conventional ERP-based speller.

### 5.3. Other hBCIs Based on Multimodal Signals

Other hybrid BCIs based on multiple signals have also been reported. A way to make full use of the spatial and temporal information of brain activity is to combine the fMRI with EEG in BCIs. A key advantage of EEG-fMRI combined BCI is that EEG can provide online slow cortical potential (SCP) feedback to subjects. It also reveals the basic psychophysiological mechanisms, such as the correlation between local BOLD-responses and the SCP changes, which helps to develop new training procedures and paradigms. Although fNIRS has poor spatial resolution compared to fMRI, it is portable and reflects the hemodynamic response of brain activity.

The authors in [45] have proved that the performance of an MI-based BCI was improved significantly by combing EEG and NIRS. It allows those who are unable to run EEG-based BCI alone to achieve meaningful classification rates. EEG is easily distorted by the inhomogeneities of the extracerebral tissues, while MEG is not affected as long as the electric inhomogeneities are concentric. Therefore, MEG signals are more local than the corresponding EEG signals and can provide more spatial information. In [47], the MEG and EEG signals generated in the sensorimotor cortex were used to index the finger movements for three tetraplegics.

## 6. Discussion and Conclusion

This paper focuses on several hBCI types and different stimulus designs and their performance analysis. To begin with, we summarized three classes of hBCIs: hBCIs based on multiple brain patterns, multisensory hBCIs, and hBCIs based on multimodal signals. For each type of hBCIs, we reviewed several representative hybrid BCI systems, including their design principles, stimuli paradigms, control methods, experimental results, and corresponding advantages. In the following, we will elaborate concluding remarks regarding the benefits of hybrid BCI systems and future studies.

Following consideration of the three types of hybrid BCI and their respective applications, we can summarize the advantages of hybrid BCI in two aspects. First, the hBCI system can provide only a single control signal or output to improve the classification performance. The two main strategies for bringing about these improvements are as follows: (1) the combination of multiple brain patterns (such as MI, P300, and SSVEP) or the fusion of multiple signals (such as EEG, EMG, EOG, and NIRS) can be performed at the feature level; and (2) enhancing brain patterns by presenting multiple sensory stimuli, such as audiovisual stimuli. Second, when multiple control signals or outputs are available, hBCI systems attempt to implement multi-degree object control. In this paper, the multi-dimensional or functional control method based on hybrid BCIs and some application systems are presented. Two main methods can be adopted: (1) combining multiple brain patterns to obtain multiple independent control signals, such as 2D cursor control based on MI and P300 and orthopedic control based on MI and SSVEP; (2) using different signal characteristics to perform different functions, such as robot control based on EEG and EOG.

Here, we consider several challenging problems for further study.

### 6.1. Design and Implementation for hBCIs

From the user's point of view, the complexity of the hBCI system is usually higher than that of the conventional simple BCI. User acceptability is an important performance criterion that needs to be carefully considered in hBCI design and implementation. In the design of an hBCI based on brain patterns, one of the challenges is how to determine the best combination of brain patterns to achieve the desired goals, and the combination can vary from user to user. For example, it should be considered that long-term use of SSVEP and P300 will increase visual fatigue. While designing a couple sensory hBCI, the challenge is to ensure that the desired brain patterns are enhanced by multiple sensory stimuli. Previous studies [33] have found that combining audio stimuli with natural spoken words in a visual P300-based BCI can help reduce the burden of mental work. Therefore, we can consider more combinations of multiple sensory stimuli involving auditory and tactile patterns in future research. For the hBCI based on multiple signals, one challenge is how to make full use of the characteristics of different signals to achieve the greatest improvement in system performance. In addition, when designing the real-time hBCI based on EEG and fMRI, the high noise, slow response and high dimensionality of EEG data (generated by fMRI scanner), and the low temporal resolution of fMRI data are not negligible.

### 6.2. Brain Mechanisms for hBCIs

The hBCI system may involve multiple brain modes, multiple sensory modes, or multimode signal inputs. To ensure that these components are effectively coordinated in the hBCI system, it is necessary to study the relevant brain mechanisms. For example, cross-modal integration/interaction in the brain can provide a brain mechanism for multisensory BCI. However, there have been few studies on the brain mechanism of hBCI so far.

### 6.3. Clinical Application

Until now, most hBCI systems (such as BCI browsers and BCI wheelchairs) were designed for healthy subjects. It needs to be extended to patients and extend their value to clinical applications. In recent years, more and more hBCIs have been used in clinical applications, such as in the rehabilitation and treatment of patients with hemiplegia [51, 52] and DOC [53]. When designing these hBCI systems for patients, the differences between them and healthy subjects need to be fully considered. In some cases, even a single patient design is necessary. The application of hBCI to patients with DOC has just begun, and hBCI-based communication and rehabilitation is an important topic for our future research. In addition, a variety of intelligent technologies, such as automatic navigation systems and intelligent robots, have been combined with BCI. This combination not only greatly reduces the user's workload but also makes the BCI system more reliable, flexible, and powerful by allowing the subject to focus on the final goal and to ignore the low-level details associated with the execution of the action. This is promising for patients with low recognition and control capabilities. Therefore, future research should focus on such systems developed for patients.

## Figures and Tables

**Table 1:** Representative hBCI applications of multiple brain patterns.

Reference	Hybrid mode	Application	Classifiers	Commands	Accuracy (%)	Improvements
[19]	SSVEP, P300, MI	Humanoid machine navigation	CCA	6	P300: 84.6, SSVEP: 84.1	Better commands performance in navigation and exploration
[20]	SSVEP, P300	Wheelchair control with stop command	SVM	2	>80	Higher detection accuracy and low response time
[21]	SSVEP, P300	Target selection speller	SW-LDA	9	93.3	More effective in target discrimination
[22]	SSVEP, P300	Cursor control	SVM	9	>90	Higher accuracy and better commands performance
[11]	SSVEP, P300	Multiple option selection	CCA, LDA	4	P300: 99.9 SSVEP: 67.2	Better performance and user-friendly
[23]	P300, SSVEP	Speller	SW-LDA	36	93.85	Higher accuracy
[24]	MI, SSVEP	Play Tetris games in MI-SSVEP paradigm	LDA, CSP, CCA	4	MI: 87.01 SSVEP: 90.26	Higher accuracy
[25]	MI, SSVEP	Hybrid BCI system of MI and SSVEP	LDC	2	85.6 ± 7.7	Better classification performance
[9]	MI, SSVEP, visual, and auditory	Wheelchair control	SVM	6	—	Multidegree control commands
[26]	MI, SSVEP	Hybrid BCI system with feedback	LDA	2	≥83	Better MI training performance
[27]	SSVEP, MI	Control commands	CCA	5	MI: 93.3 SSVEP: 89	Better performance and easiness for users
[16]	MI, P300	2-D cursor control	SVM	2	>80	Multiple-degree control
[17]	P300, MI	BCI mouse-based web browser	SVM	3	93.21	Multidegree control with a feasible BCI mouse
[28]	P300, MI	BCI wheelchair with direction and speed control	LDA	4	83.10 ± 2.12	Direction and speed control

**Table 2:** Representative applications of multisensory hBCIs.

Reference	Hybrid mode	Application	Classifiers	Commands	Accuracy (%)	Improvements
[30]	P300, visual, audio	P300 audiovisual speller	Regularized linear LR	—	>80	Improvement in performance
[31]	Visual, audio	Consciousness detection in patients with DOC	SVM	2	>64	Better performance and feasible to patients with DOC
[32]	Visual, audio	Visual-auditory speller	LDA	30	87.7 (chance level <3%)	Better BCI performance
[33]	Visual, audio	Awareness detection	SVM	2	95.67	Better performance over auditory-only and visual-only systems
[34]	Auditory, tactile, visual, P300	Visual saccade-independent BCI	BLDA	4	88.67	Better online performance
[35]	Auditory, tactile, P300	Tactile and bone-conduction BCI	SW-LDA	6	70	Higher classification accuracy
[36]	Audio, tactile	Robot gesture	FGMMs, SVM	10	92.75	Better performance over framework

**Table 3:** Representative applications of hBCI of multimodal signals.

Reference	Hybrid mode	Application	Classifiers	Commands	Accuracy (%)	Improvements
[37]	EMG, EEG	A motor imagery hybrid BCI speller	GMM	2	End-users: 91 Able-bodied users: 94	Better performance over command accuracy
[38]	EEG, EMG	Home environmental control system	CCA	4	96.3	Higher control accuracy, security, and interactivity
[39]	EEG, EOG	AIDS recovery	AR	4	62.28	Substantially better control over assistive devices
[40]	EEG, EOG	Mobile robot control	LDA	9	87.3	Reduce the best completion time
[41]	EEG, EOG	Hybrid speller system	LDA	1	97.6	Better performance and usability
[42]	fNIRS, EEG, eye movement	Control a quadcopter online	LDA	8	fNIRS: 75.6 EEG: 86	Higher accuracy on decoding
[43]	EEG, fNIRS	Hand movement and recognition	LDA	2	94.2	Reduce fNIRS delay time in detection
[44]	EEG, fNIRS	Left- and right-hand motion imagination	DL	2	—	Reduce response time
[45]	EEG, NIRS	Decoding of four movements	LDA	5	>80	Higher classification accuracy
[46]	EEG, NIRS	Mental state recognition	Meta	6	65.6	Better performance on mental states classification
[47]	EEG, MEG	Left- and right-hand motor imagery	CSP, LR	2	MEG: 70.6 EEG: 67.7	Better performance over good within-subject accuracy
[48]	EEG, NIRS	Classification of mental arithmetic, MI, and idle state	sLDA	3	82.2 ± 10.2	Higher classification accuracy
[49]	EEG, MEG	Intersubject decoding of left- vs. right-hand motor imagery	LR, L2, 1-norm regularization	4	MEG: 70 EEG: 67.7	Higher within-subject accuracy

## References

[1] Wolpaw J. R., Birbaumer N., McFarland D. J., Pfurtscheller G., Vaughan T. M. (2002). Brain-computer interfaces for communication and control. *Clinical Neurophysiology*.

[2] Fazli S., Mehnert J., Steinbrink J. (2012). Enhanced performance by a hybrid NIRS-EEG brain computer interface. *NeuroImage*.

[3] Farwell L. A., Donchin E. (1988). Talking off the top of your head: toward a mental prosthesis utilizing event-related brain potentials. *Electroencephalography and Clinical Neurophysiology*.

[4] Müllerputz G. R., Scherer R., Neuper C., Pfurtscheller G. (2006). Steady-state somatosensory evoked potentials: suitable brain signals for brain-computer interfaces?. *IEEE Transactions on Neural Systems and Rehabilitation Engineering*.

[5] Pfurtscheller G., Lopes da Silva F. H. (1999). Event-related EEG/MEG synchronization and desynchronization: basic principles. *Clinical Neurophysiology*.

[6] Hong K. S., Khan M. J. (2017). Hybrid brain–computer interface techniques for improved classification accuracy and increased number of commands: a review. *Frontiers in Neurorobotics*.

[7] Vučković A., Sepulveda F. (2012). A two-stage four-class BCI based on imaginary movements of the left and the right wrist. *Medical Engineering & Physics*.

[8] Allison B. Z., Graimann B., Pfurtscheller G., Allison B. (2010). Toward ubiquitous BCIs. *Brain-Computer Interfaces: Revolutionizing Human-Computer Interaction*.

[9] Li J., Ji H., Cao L. (2014). Evaluation and application of a hybrid brain computer interface for real wheelchair parallel control with multi-degree of freedom. *International Journal of Neural Systems*.

[10] Pfurtscheller G., Allison B. Z., Brunner C. (2010). The hybrid BCI. *Frontiers in Neuroscience*.

[11] Allison B. Z., Jin J., Zhang Y., Wang X. (2014). A four-choice hybrid P300/SSVEP BCI for improved accuracy. *Brain-Computer Interfaces*.

[12] Wagner I. C., Daly I., Väljamäe A. (2012). Non-visual and Multisensory BCI Systems: Present and Future. *Towards Practical Brain-Computer Interfaces*.

[13] Panicker R. C., Puthusserypady S., Sun Y. (2011). An asynchronous P300 BCI with SSVEP-based control state detection. *IEEE Transactions on Biomedical Engineering*.

[14] Li Y., Pan J., Long J. (2016). Multimodal BCIs: target detection, multidimensional control, and awareness evaluation in patients with disorder of consciousness. *Proceedings of the IEEE*.

[15] Pfurtscheller G., Solis-Escalante T., Ortner R., Linortner P., Muller-Putz G. R. (2010). Self-paced operation of an SSVEP-based orthosis with and without an imagery-based “brain switch:” a feasibility study towards a hybrid BCI. *IEEE Transactions on Neural Systems and Rehabilitation Engineering*.

[16] Li Y., Long J., Yu T. (2010). An EEG-based BCI system for 2-D cursor control by combining mu/beta rhythm and P300 potential. *IEEE Transactions on Biomedical Engineering*.

[17] Yu T., Li Y., Long J., Gu Z. (2012). Surfing the internet with a BCI mouse. *Journal of Neural Engineering*.

[18] Yu T., Li Y., Long J., Li F. (2013). A hybrid brain-computer interface-based mail client. *Computational and Mathematical Methods in Medicine*.

[19] Choi B., Jo S. (2013). A low-cost EEG system-based hybrid brain-computer interface for humanoid robot navigation and recognition. *PLoS One*.

[20] Li Y., Pan J., Wang F., Yu Z. (2013). A hybrid BCI system combining P300 and SSVEP and its application to wheelchair control. *IEEE Transactions on Biomedical Engineering*.

[21] Xu M., Qi H., Wan B., Yin T., Liu Z., Ming D. (2013). A hybrid BCI speller paradigm combining P300 potential and the SSVEP blocking feature. *Journal of Neural Engineering*.

[22] Bi L., Lian J., Jie K., Lai R., Liu Y. (2014). A speed and direction-based cursor control system with P300 and SSVEP. *Biomedical Signal Processing and Control*.

[23] Yin E., Zeyl T., Saab R., Chau T., Hu D., Zhou Z. (2015). A hybrid brain-computer interface based on the fusion of P300 and SSVEP scores. *IEEE Transactions on Neural Systems and Rehabilitation Engineering*.

[24] Wang Z., Yu Y., Xu M., Liu Y., Yin E., Zhou Z. (2019). Towards a hybrid BCI gaming paradigm based on motor imagery and SSVEP. *International Journal of Human-Computer Interaction*.

[25] Ko L.-W., Ranga S. S. K., Komarov O., Chen C.-C. (2017). Development of single-channel hybrid BCI system using motor imagery and SSVEP. *Journal of Healthcare Engineering*.

[26] Yu T., Xiao J., Wang F. (2015). Enhanced motor imagery training using a hybrid BCI with feedback. *IEEE Transactions on Biomedical Engineering*.

[27] Duan F., Lin D., Li W., Zhang Z. (2015). Design of a multimodal EEG-based hybrid BCI system with visual servo module. *IEEE Transactions on Autonomous Mental Development*.

[28] Long J., Li Y., Wang H., Yu T., Pan J., Li F. (2012). A hybrid brain computer interface to control the direction and speed of a simulated or real wheelchair. *IEEE Transactions on Neural Systems and Rehabilitation Engineering*.

[29] Pan J., Xie Q., He Y. (2014). Detecting awareness in patients with disorders of consciousness using a hybrid brain–computer interface. *Journal of Neural Engineering*.

[30] Belitski A., Farquhar J., Desain P. (2011). P300 audio-visual speller. *Journal of Neural Engineering*.

[31] Pan J., Xie Q., Huang H. (2018). Emotion-related consciousness detection in patients with disorders of consciousness through an EEG-based BCI system. *Frontiers in Human Neuroscience*.

[32] An X., Höhne J., Ming D., Blankertz B. (2014). Exploring combinations of auditory and visual stimuli for gaze-independent brain-computer interfaces. *PLoS One*.

[33] Wang F., He Y., Pan J. (2015). Erratum: a novel audiovisual brain-computer interface and its application in awareness detection. *Scientific Reports*.

[34] Yin E., Zeyl T., Saab R., Hu D., Zhou Z., Chau T. (2016). An auditory-tactile visual saccade-independent P300 brain-computer interface. *International Journal of Neural Systems*.

[35] Rutkowski T. M., Mori H. (2015). Tactile and bone-conduction auditory brain computer interface for vision and hearing impaired users. *Journal of Neuroscience Methods*.

[36] Ju Z., Liu H. (2014). Human hand motion analysis with multisensory information. *IEEE/ASME Transactions on Mechatronics*.

[37] Perdikis S., Leeb R., Williamson J. (2014). Clinical evaluation of BrainTree, a motor imagery hybrid BCI speller. *Journal of Neural Engineering*.

[38] Chai X., Zhang Z., Lu Y., Liu G., Zhang T., Niu H. (2019). A hybrid BCI-based environmental control system using SSVEP and EMG signals. *World Congress on Medical Physics and Biomedical Engineering 2018*.

[39] Soekadar S. R., Witkowski M., Vitiello N., Birbaumer N. J. B. E. (2015). An EEG/EOG-based hybrid brain-neural computer interaction (BNCI) system to control an exoskeleton for the paralyzed hand. *Biomedical Engineering*.

[40] Ma J., Zhang Y., Cichocki A., Matsuno F. (2015). A novel EOG/EEG hybrid human-machine interface adopting eye movements and ERPs: application to robot control. *IEEE Transactions on Biomedical Engineering*.

[41] Lee M. H., Williamson J., Won D. O., Fazli S., Lee S. W. (2018). A high performance spelling system based on EEG-EOG signals with visual feedback. *IEEE Transactions on Neural Systems and Rehabilitation Engineering*.

[42] Khan M. J., Hong K. S. (2017). Hybrid EEG-fNIRS-based eight-command decoding for BCI: application to quadcopter control. *Frontiers in Neurorobotics*.

[43] Buccino A. P., Keles H. O., Omurtag A. (2016). Hybrid EEG-fNIRS asynchronous brain-computer interface for multiple motor tasks. *PLoS One*.

[44] Chiarelli A. M., Croce P., Merla A., Zappasodi F. (2018). Deep learning for hybrid EEG-fNIRS brain-computer interface: application to motor imagery classification. *Journal of Neural Engineering*.

[45] Khan M. J., Hong M. J., Hong K. S. J. FiH. N. (2014). Decoding of four movement directions using hybrid NIRS-EEG brain-computer interface. *Frontiers in Human Neuroscience*.

[46] Shin J., von Luhmann A., Blankertz B. (2017). Open access dataset for EEG+NIRS single-trial classification. *IEEE Transactions on Neural Systems and Rehabilitation Engineering*.

[47] Halme H. L., Parkkonen L. (2018). Across-subject offline decoding of motor imagery from MEG and EEG. *Scientific Reports*.

[48] Shin J., Kwon J., Im C.-H. (2018). A ternary hybrid EEG-NIRS brain-computer interface for the classification of brain activation patterns during mental arithmetic, motor imagery, and idle state. *Frontiers in Neuroinformatics*.

[49] Halme H.-L., Parkkonen L. (2018). Across-subject offline decoding of motor imagery from MEG and EEG. *Scientific Reports*.

[50] Leeb R., Sagha H., Chavarriaga R., del R Millán J. (2011). A hybrid brain-computer interface based on the fusion of electroencephalographic and electromyographic activities. *Journal of Neural Engineering*.

[51] Hassan M., Kadone H., Ueno T., Hada Y., Sankai Y., Suzuki K. (2018). Feasibility of synergy-based exoskeleton robot control in hemiplegia. *IEEE Transactions on Neural Systems and Rehabilitation Engineering*.

[52] Kato K., Takahashi K., Mizuguchi N., Ushiba J. (2018). Online detection of amplitude modulation of motor-related EEG desynchronization using a lock-in amplifier: comparison with a fast Fourier transform, a continuous wavelet transform, and an autoregressive algorithm. *Journal of Neuroscience Methods*.

[53] Wang F., He Y., Qu J. (2017). Enhancing clinical communication assessments using an audiovisual BCI for patients with disorders of consciousness. *Journal of Neural Engineering*.

